# Network Analyses Applied to the Dimensions of Cancer‐Related Fatigue in Women With Breast Cancer

**DOI:** 10.1002/cam4.70268

**Published:** 2024-10-10

**Authors:** Louise Baussard, Marie Ernst, Anh Diep, Guy Jerusalem, Audrey Vanhaudenhuyse, Nolwenn Marie, Isabelle Bragard, Marie‐Elisabeth Faymonville, Olivia Gosseries, Charlotte Grégoire

**Affiliations:** ^1^ Epsylon Laboratory University Paul Valéry Montpellier 3 Nîmes France; ^2^ Biostatistics and Research Methods Centre University Hospital and University of Liège Liège Belgium; ^3^ Medical Oncology Department University Hospital and University of Liège Liège Belgium; ^4^ Interdisciplinary Algology Centre University Hospital of Liège Liège Belgium; ^5^ Sensation and Perception Research Group GIGA‐Consciousness, University of Liège Liège Belgium; ^6^ Research and Continuing Education Department, CRIG Research Center Haute Ecole Libre Mosane (HELMo) Liège Belgium; ^7^ Arsène Burny Cancerology Institute, University Hospital of Liège Liège Belgium; ^8^ Coma Science Group, GIGA‐Consciousness University of Liège Liège Belgium; ^9^ Centre du Cerveau² University Hospital of Liège Liège Belgium

**Keywords:** breast cancer, cancer‐related fatigue, network analysis, psychoneurological symptom cluster

## Abstract

**Background:**

Understanding cancer symptom cluster through network analyses is a new approach in oncology, revealing interconnected and influential relationships among reported symptoms. We aimed to assess these relationships using network analysis in posttreatment breast cancer patients, focusing on the five dimensions of cancer‐related fatigue (CRF), and on other common difficulties encountered by oncological patients (i.e., pain, anxiety, depression, sleep difficulties, cognitive impairments, and emotion regulation and mental adaptation difficulties).

**Method:**

This study involved a complementary analysis of data from two interventional studies. Participants completed questionnaires before and after the intervention, with baseline scores being used in this article. Partial correlation network analysis modeled the relationships between symptoms in five distinct networks, each of them including one specific dimension of CRF. The core symptom in each network was identified based on the highest centrality indices.

**Results:**

Depression emerged as the core symptom in all networks, strongly associated with all fatigue dimensions (partial correlations ranging from 0.183 to 0.269) except mental fatigue. These findings indicate robust connections between symptoms, as variations in depression scores directly or indirectly influence fatigue and other symptoms within the cluster.

**Conclusion:**

Our results support the multidimensional aspect of CRF, and its links with other common symptoms. To effectively reduce patient CRF, interventions should address not only fatigue but also the closely related symptoms from the cluster, such as depression, given its centrality in the cluster.

**Trial Registration:** ClinicalTrials.gov (NCT03144154 and NCT04873661). Retrospectively registered on May 1, 2017 and April 29, 2021, respectively.

## Graphical abstract

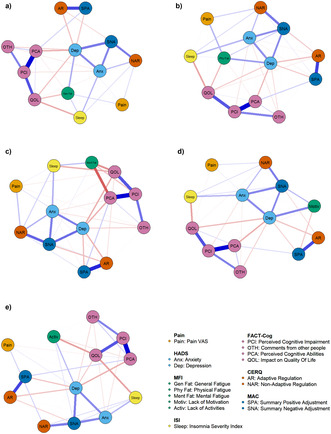

## Introduction

Cancer‐related fatigue (CRF) is defined as a distressing, persistent, and subjective feeling of physical, emotional, and/or cognitive tiredness or exhaustion, which is related to cancer or its treatment. It is not proportional to the person's recent activity and interferes with their usual functioning [1]. CRF is estimated to be endured by 66% of survivors of breast cancer [2], and considered as the most severe symptom reported by these women, with a high impact on their quality of life [3]. CRF is a multidimensional symptom, frequently divided into five main dimensions: general fatigue (i.e., general functional state), physical fatigue (i.e., physical sensation linked to the feeling of tiredness), mental fatigue (i.e., cognitive symptoms such as a lack of concentration), lack of motivation and lack of activity (i.e., reduction in activities and lack of motivation to start any activity) [4]. These dimensions seem to evolve differently over time [5, 6, 7], to have different determinants (e.g., neuroticism, relationship status, optimism, treatments received, obesity, social support), [5, 6, 7, 8] and to respond differently to various interventions (e.g., physical exercise, acupressure, acupuncture, cognitive–behavioral therapy) [7, 9, 10], supporting the multidimensional aspect of CRF.

CRF is part of a larger psychoneurological symptom cluster (PNSC), also comprising emotional distress (i.e., anxiety and depression), sleep difficulties, and pain—that is increasingly documented in oncology—notably in women with breast cancer [11, 12, 13, 14]. Cognitive impairments are also frequently associated with this symptom cluster [15, 16]. In addition to their own negative consequences, these symptoms are known to evolve together and to reinforce each other, participating in the high burden endured by the patients [11, 17]. The mechanisms involved in the PNSC as well as the relationships between its symptoms are still unclear. However, despite their severe impact and their persistence up to years after treatment completion, these symptoms remain underdiagnosed and undertreated [18]. Other difficulties linked to coping are also frequently reported by women with breast cancer, such as maladaptive coping strategies and difficulties in emotion regulation [19, 20, 21]. Maladaptive emotion regulation and coping strategies are known to be linked with increased depression and anxiety, and decreased quality of life [21, 22, 23].

Most studies in oncology focused on a single symptom, or on several symptoms considered independently from each other [9, 12, 24, 25, 26, 27]. However, the high prevalence of the PNSC underlines the relevance of studying multiple symptoms and their interactions, for example, through network analyses. By assessing and visualizing symptom clusters as dynamic systems of interacting symptoms, it allows to study them in their full complexity, in line with the patient's clinical reality [11, 14, 17, 28]. Core symptoms within a network are the ones with the strongest associations with the other symptoms and which may play a critical role in activating them [29]. Identifying them is crucial, as they could allow a better understanding of the burden endured by patients with cancer, and represent a relevant target to impact the whole cluster through different interventions [11, 12, 14, 25, 27, 28, 30, 31].

As network analyses have been very seldom applied in oncology, there is no consensus regarding the core symptom of the PNSC yet. It seems to vary according to the population studied, the phase of the cancer trajectory, and the methodology used. Some previous network analyses performed on women with breast cancer suggested that emotional symptoms (i.e., irritability, mood swings) [32] or CRF [11] could be the core symptom. When women with breast cancer are considered altogether with patients with other cancer diagnoses, depressive symptoms [27], anxiety [31], or CRF [14] have been suggested as core symptoms. Due to the sparsity and heterogeneity of literature on symptom clusters in oncology, more rigorous studies are needed to assess the presence and configuration of the PNSC among women with breast cancer, especially in regard to the main dimensions of CRF. In this context, network analyses represent an interesting and relevant tool to improve the understanding of the PNSC and more specifically CRF.

We aim to assess and visualize the relationships between the different symptoms from the PNSC, considered in their different dimensions, in a large population of posttreatment breast cancer patients. Our focus will be CRF, considered in its five dimensions (i.e., general, physical and mental fatigue, reduced activity, and reduced motivation). Thus, five distinct networks will be constructed to assess the relationships between each CRF dimension and all the other symptoms. We hypothesize that, in each network, the CRF dimension considered, or emotional symptoms such as depression or anxiety, will be the core symptom. We also hypothesize that the relationships between the CRF dimension and other symptoms will be different between the five networks.

## 1 Materials and Methods

### 1.1 Design

This study is a secondary analysis of data from two longitudinal interventional studies conducted by our team. Their respective detailed protocols have been published [33, 34]. The first study [34] was a randomized controlled trial including patients with different cancers, conducted between 2017 and 2020. It aimed to assess the benefits of a hypnosis‐based group intervention on their quality of life. The second study [33] started in 2021 and was ongoing when writing this manuscript (recruitment completed in May 2024). It is a preference‐based trial aiming at exploring the benefits of three group interventions based on non‐ordinary states of consciousness (i.e., hypnosis, mindful self‐compassion meditation, and auto‐induced cognitive trance) on the quality of life of patients with different cancers. In both studies, participants had to complete several questionnaires before and after the intervention. Some of these questionnaires were used in both studies. Only their baseline data were considered in this article.

### 1.2 Participants

Participants were recruited between 2017 and 2023, mainly at the University Hospital of Liège (Belgium) but also in other structures and through social media, in the context of two other studies [33, 34]. The cohort analyzed in this article is thus composed of women with breast cancer who participated in one of these two studies. We chose to focus on women with breast cancer because they represented the most part of the studies' samples. All the women considered in this article met the following inclusion criteria: ≥ 18‐year‐old, diagnosis of breast cancer (metastatic cancers included), being fluent in French, all active treatments (i.e., surgery, chemotherapy, radiation therapy) completed for less than 1 year, presence of emotional or physical difficulties at baseline, as established by a score of at least 4 out of 10 on one of the following symptoms: physical fatigue, mental fatigue, anxiety, depression, sleep difficulties, pain, ruminations, or fear of recurrence. They had to sign an informed consent before participating in their respective study. This cut‐off score was chosen to avoid floor effects [35]. As the present cohort came from previous studies of our team, no sample size was calculated specifically for this work. In addition, it seems that no standard procedure to determine the ideal sample size for network analysis is commonly used [11, 12, 28, 30]. The first study included 104 patients with cancer, of which 75 were women with breast cancer. Our second study is still ongoing and at the present moment, 115 participants are included, of which 84 are women with breast cancer. Altogether, these two samples lead to a final sample of 159 women with breast cancer.

### 1.3 Assessments

Several questionnaires were completed before participating in the interventions proposed in the studies. In the first study, participants completed a paper form and in the second study, they completed the questionnaires online.

- *General information*: Sociodemographic and medical data (age, education level, marital situation, cancer diagnosis, time since diagnosis, and treatments received) have been collected.
- *Symptoms from the PNSC*:
  ○ *Pain—Visual Analog Scale* (*VAS*): The VAS assessed the pain level of the participant during the last week, with scores comprised between 0 (no pain) and 10 (worst pain ever).
  ○ *Multidimensional Fatigue Inventory* (*MFI‐20*) [4, 36]: This scale covers the five fatigue dimensions already detailed: general, physical, and mental fatigue, as well as reduced motivation and activity. Each dimension scores from 4 to 20, with higher scores indicating higher fatigue, and less motivation or activities. In women aged between 40 and 59, a score ≥ 12 on the general fatigue subscale suggests significant fatigue [37, 38].
  ○ *Insomnia Severity Index* (*ISI*) [39]: This scale investigates the participant's sleep complaints and the distress associated. Scores range from 0 to 28, with a score between 0 and 7 suggesting the absence of insomnia, a score between 8 and 14 suggesting subclinical insomnia, a score between 15 and 21 suggesting moderate insomnia, and a score over 22 suggesting severe insomnia.
  ○ *Hospital Anxiety and Depression Scale* (*HADS*) [40]: This questionnaire measures anxiety and depression. Each dimension scores from 0 to 21, with higher scores indicating higher probability of anxious or depressive disorders. More precisely, a score between 0 and 7 indicates the absence of anxious or depressive disorder, a score between 8 and 10 indicates suspected anxious or depressive disorder, and a score over 11 indicates the presence of anxious or depressive disorder.
  ○ *Functional Assessment of Cancer Therapy—Cognitive Function* (*FACT‐Cog v.3*) [41]: This questionnaire measures the participant's subjective cognitive functioning over the past week. It is composed of four subscales: perceived cognitive impairments (PCI), comments from other people regarding cognitive difficulties (OTH), perceived cognitive abilities (PCA), and the impact of perceived cognitive impairments on quality of life (QOL). Each dimension scores from 0 to 72, 16, 28, and 16, respectively, with higher scores indicating less cognitive impairments, less comments from other people, more cognitive abilities, and less impact on quality of life, respectively.
- *Other symptoms*:
  ○ *Mental Adjustment to Cancer Scale* (*MAC*) [42]: This questionnaire assesses the participant's coping styles and adjustment to cancer and is divided into two subscales: summary positive adjustment (SPA, ranging from 17 to 68) and summary negative adjustment (SNA, ranging from 16 to 64). A higher score indicates a higher positive or negative adjustment.
  ○ *Cognitive Emotion Regulation Questionnaire* (*CERQ*) [43]: This questionnaire investigates the cognitive emotion regulation strategies used by the participant after experiencing negative events linked with the disease or its treatments. It has two main subscales: adaptive emotion regulation (ranging from 20 to 100) and nonadaptive emotion regulation (ranging from 16 to 80), with higher scores indicating higher adaptive or nonadaptive emotion regulation.

### 1.4 Data Analyses

All statistical analyses were performed using SAS software (version 9.4) and R software (version 4.2.2), more precisely the R packages *qgraph* and *bootnet* [44]. Descriptive statistics were used to describe the sample (mean and standard deviations [SD] for quantitative variables, and number and percentages for categorical variables). Partial (Pearson) correlation network was used to model the conditional independence relationship between symptoms (nodes of the networks). In order to add sparsity in the networks, a regularized Lasso estimation was used where the tuning parameter was chosen based on a BIC. Sparse networks are presented in this article. The centrality indices used were the strength (number and strength of the direct connections to a node/symptom, i.e., sum of absolute weights), the closeness (node's relationship to all other nodes), and the betweenness (importance of a node in the average pathway between other pairs of nodes). The core symptom in each network was identified based on the highest centrality indices. Bootstrapping (nBoots = 1000) was used to evaluate the accuracy of the networks, providing estimations and confidence intervals (CI) for node's strength and edge weights. Partial correlations were considered to be significant at *p* < 0.05.

## 2 Results

### 2.1 Description of the Sample

Table 1 details the sociodemographic and medical data of the sample. Women in the sample had a mean age of 51.8 (SD = 9.8) years and were mostly living with a partner (*n* = 103, 64.8%). Mean time since diagnosis was 13.3 months (SD = 12.8). The high range of this variable is likely to be linked to the fact that four of them were facing a cancer recurrence (local recurrence: *N* = 1; distant recurrence/metastases: *N* = 3) at the time of their inclusion in the study. Most women of the sample had several modalities of treatments, especially surgery, radiation therapy, and hormonal therapy. Table 2 describes the clinical symptoms reported by the women in our sample.

**TABLE 1:** Sociodemographic and medical data of the sample (*N* = 159).

Variable	Mean (SD)
	Range
Age (years)	51.8 (9.8) 30–79
Time since diagnosis (months)	13.3 (12.8) 2–135
Variable	N (%)
Marital status	
Single	15 (9.4)
Married/living with partner	103 (64.8)
Divorced/separated/widowed	27 (17.0)
In a relationship but not living together	14 (8.8)
Education level	
Lower secondary school	9 (5.7)
Upper secondary school	32 (20.1)
Bachelor's degree	58 (36.5)
Master's degree	54 (34.0)
Postgraduate	6 (3.8)
Surgery	
Yes	157 (98.7)
No	1 (0.6)
Missing data	1 (0.6)
Chemotherapy (CT)	
Yes	95 (69.7)
No	60 (37.7)
Missing data	4 (2.5)
Radiation therapy (RT)	
Yes	139 (87.4)
No	19 (11.9)
Missing data	1 (0.6)
Hormonal therapy (HT)	
Yes	120 (75.5)
No	34 (21.4)
Missing data	5 (3.1)

**TABLE 2:** Clinical symptoms reported in the sample (*N* = 159).

Variable	Mean (SD)
Pain (VAS 0–10)	4.5 (2.4)
Multidimensional Fatigue Inventory (MFI‐20)	
General fatigue	15.4 (3.2)
Physical fatigue	13.6 (3.5)
Mental fatigue	13.0 (3.9)
Lack of motivation	9.7 (3.3)
Lack of activities	11.8 (3.5)
Hospital Anxiety and Depression Scale (HADS)	
Anxiety	9.9 (4.1)
Depression	6.1 (3.8)
Insomnia Severity Index (ISI)	
Sleep difficulties	14.3 (6.3)
Functional Assessment of Cancer Therapy‐Cognitive Function (FACT‐Cog)	
Perceived cognitive impairment (PCI)	42.1 (15.8)
Comments from other people (OTH)	14.1 (3.1)
Perceived cognitive abilities (PCA)	14.2 (5.4)
Impact on quality of life (QOL)	8.1 (4.5)
Cognitive Emotion Regulation Questionnaire (CERQ)	
Adaptive emotion regulation	70.8 (13.7)
Nonadaptive emotion regulation	31.0 (8.8)
Mental Adjustment to Cancer (MAC)	
Summary positive adjustment (SPA)	49.2 (5.7)
Summary negative adjustment (SNA)	32.9 (7.8)

### 2.2 Network Analyses Applied to the Five Dimensions of CRF

**FIGURE 1:**
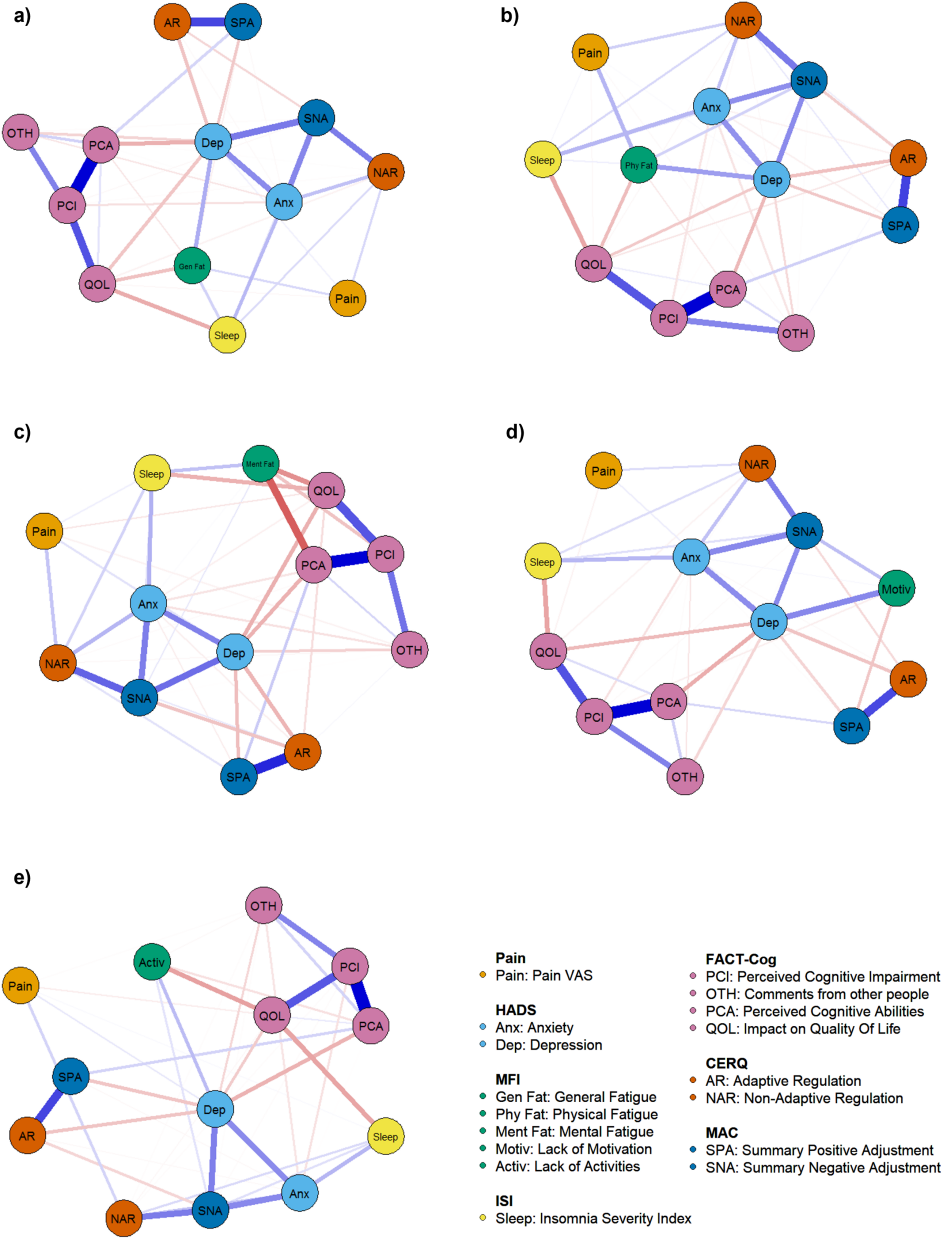
Symptom networks associated with the five dimensions of CRF: (a) general fatigue, (b) physical fatigue, (c) mental fatigue, (d) lack of motivation, and (e) lack of activities. Blue edges indicate positive relationships, red edges indicate negative relationships. Thicker edges indicate stronger relationships (i.e., higher partial correlation).

Table 3 details the partial correlation and 95% CI between each fatigue dimension and the other symptoms. In addition, Figure 1 illustrates the five associated sparse networks. Detailed weights are provided in Table S1. Note that bootstrap procedures were used to confirm the network accuracy (see Figure S1). Depression is significantly associated with each CRF dimension (*r* ranging from 0.183 to 0.269) except the mental fatigue. Considering all the other symptoms, general fatigue is also significantly positively connected to pain (*r* = 0.176) and sleep difficulties (*r* = 0.187), and negatively connected to nonadaptive emotion regulation (*r* = −0.203). Physical fatigue is significantly positively connected to pain (*r* = 0.221) and negatively to mental adjustment to cancer (SNA; *r* = 0.209). Mental fatigue is significantly positively correlated to sleep difficulties (*r* = 0.192) and negatively to FACT‐Cog PCA (*r* = −0.358) and QOL (*r* = −0.216). As these two FACT‐Cog subscales are reversely scored, these results suggest that a higher mental fatigue is linked to a higher perceived cognitive impairment and to a higher impact of these difficulties on QOL. Lack of motivation is significantly associated with SNA (*r* = 0.156). Finally, lack of activities is significantly positively correlated with SNA (*r* = 0.171) and negatively with anxiety (*r* = −0.200) and FACT‐Cog QOL (*r* = −0.231). Note that the partial correlations between two “nonfatigue” symptoms (i.e., pain VAS, HADS, ISI, FACT‐Cog, CERQ, and MAC scales) are quite constant in all networks (i.e., significant/nonsignificant links remain significant/nonsignificant in all networks; see Table S1). In all five networks, SNA is positively associated with depression, anxiety, and CERQ nonadaptive regulation, while SPA is strongly associated with CERQ adaptive regulation. In the general fatigue network, depression is also positively related to SPA and anxiety, and negatively related to FACT‐Cog PCA.

**TABLE 3:** Partial correlations and 95% confidence intervals (CI) between each CRF dimension and the other symptoms.

Partial correlation and 95% CI (%)	General fatigue		Physical fatigue		Mental fatigue		Lack of motivation		Lack of activities	
	*r*	95% CI	*r*	95% CI	*r*	95% CI	*r*	95% CI	*r*	95% CI
Pain VAS	**0.176**	**0.021**; **0.323**	**0.221**	**0.067**; **0.364**	−0.089	−0.242; 0.067	0.050	−0.106; 0.204	0.016	−0.140; 0.171
HADS anxiety	−0.052	−0.206; 0.105	−0.149	−0.298; 0.006	0.024	−0.132; 0.179	−0.004	−0.160; 0.151	**−0.200**	**−0.345**; **−0.046**
HADS depression	**0.192**	**0.038**; **0.338**	**0.251**	**0.099**; **0.391**	0.007	−0.149; 0.163	**0.269**	**0.118**; **0.407**	**0.183**	**0.029**; **0.330**
ISI sleep difficulties	**0.187**	**0.032**; **0.333**	0.119	−0.037; 0.270	**0.192**	**0.038**; **0.338**	0.106	−0.051; 0.257	−0.011	−0.166; 0.145
FACT‐Cog PCI	−0.011	−0.166; 0.145	−0.013	−0.168; 0.143	−0.087	−0.240; 0.069	−0.095	−0.247; 0.061	0.041	−0.115; 0.196
FACT‐Cog OTH	0.094	−0.063; 0.246	0.020	−0.136; 0.176	0.056	−0.100; 0.210	0.028	−0.128; 0.183	−0.086	−0.238; 0.071
FACT‐Cog PCA	−0.071	−0.224; 0.086	−0.057	−0.211; 0.099	**−0.358**	**−0.486**; **−0.214**	0.031	−0.125; 0.186	−0.072	−0.225; 0.085
FACT‐Cog QOL	−0.131	−0.281; 0.025	−0.127	−0.277; 0.029	**−0.216**	**−0.359**; **−0.062**	0.073	−0.084; 0.226	**−0.231**	**−0.373**; **−0.078**
CERQ adaptive regulation	0.000	−0.156; 0.156	0.084	−0.073; 0.237	−0.038	−0.192; 0.119	0.021	−0.135; 0.176	0.124	−0.032; 0.274
CERQ nonadaptive regulation	**−0.203**	**−0.347**; **−0.048**	−0.142	−0.291; 0.014	−0.080	−0.233; 0.076	−0.092	−0.244; 0.064	−0.003	−0.159; 0.153
MAC SPA	−0.063	−0.217; 0.093	−0.026	−0.181; 0.130	0.155	−0.001; 0.303	−0.149	−0.298; 0.007	−0.097	−0.249; 0.060
MAC SNA	0.085	−0.072; 0.238	**0.209**	**0.055**; **0.353**	0.095	−0.062; 0.247	**0.156**	**0.001**; **0.305**	**0.171**	**0.016**; **0.318**

*Note:* Bold values indicate significant associations.

**FIGURE 2:**
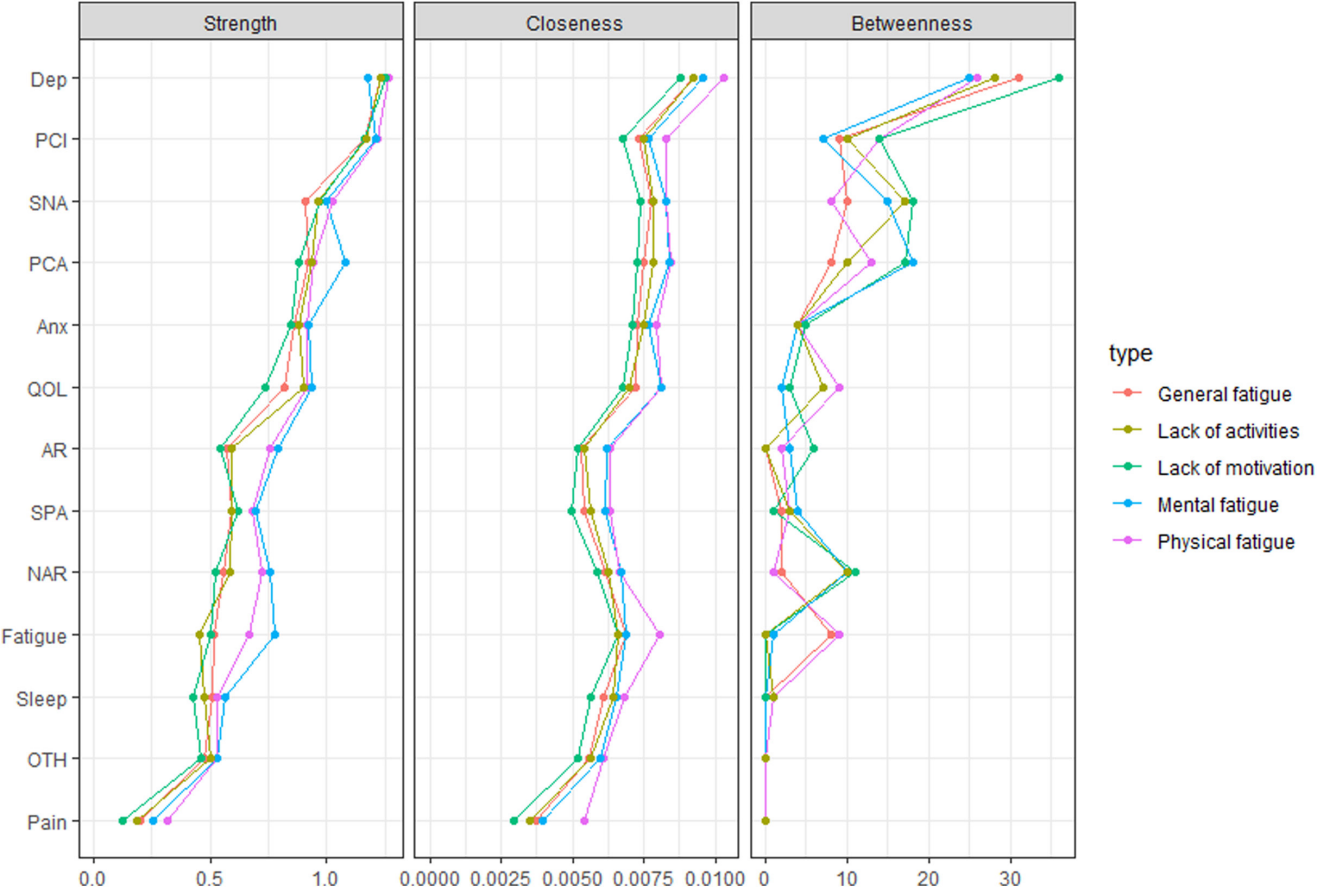
Centrality indices for the different symptoms within each network.

Figure 2 illustrates the centrality indices for the symptom networks (also see Table S2). Based on these indices, depression showed the highest strength (i.e., highest number and strength of direct connections; *r*_strength_ = 1.23–1.27) in each network (except for the network associated with the mental fatigue dimension, in which PCI has the highest strength, i.e., *r*_strength_ = 1.21), as well as the highest closeness (*r*_closeness_ = 0.00088–0.00103) and betweenness (*r*_betweenness_ = 25–36). The centrality of each symptom is similar in the five networks, and depression is the most core symptom, followed by PCI (especially in the network associated with mental fatigue), in each network. However, the centrality of each dimension of fatigue differs. For example, physical and mental fatigue have larger strength than the three other dimensions, in their respective network. Finally, pain has the smallest strength, closeness, and betweenness in each network. These results were confirmed by bootstrap procedures (also see Figure S2).

## 3 Discussion

The aim of this study was to investigate the relationships between various symptoms (PNSC) in a cohort of breast cancer survivors, focusing more specifically on the five dimensions of CRF (i.e., general fatigue, physical fatigue, mental fatigue, lack of motivation, and lack of activity). Using network analyses, we aimed to explore the relationships between different common and severe symptoms endured by these women, while emphasizing the multidimensional aspect of CRF. Our first hypothesis was that, in each network, the CRF dimension considered, or an emotional symptom such as depression or anxiety would be the core symptom. Our second hypothesis was that the relationships between the CRF dimension and other symptoms would be different between the five networks.

Our first hypothesis is verified since depression emerged as a central symptom across all five estimated networks, regardless of the CRF dimension considered. This confirms recent symptom network analysis studies involving women with breast cancer [12, 27, 32]. This is also in line with studies on patients with head and neck or gastric cancers, where depression, as well as sadness and distress, respectively, were centrally embedded in networks composed of other common symptoms [28, 30]. Our second hypothesis is also confirmed. Indeed, each CRF dimension showed specific relationships with other symptoms, as described in Section 4.2. One important finding is that depression is strongly associated with all dimensions of CRF, except mental fatigue. This can be understood by the fact that general fatigue, physical fatigue, reduced activity, and reduced motivation are known to be related to depressive symptomatology [45]. Moreover, the link between fatigue and depression has been largely documented in oncology [46] and in other clinical populations [47, 48]. The absence of significant association between depression (i.e., core symptom of the cluster) and mental fatigue is more surprising. Mental fatigue is however particularly linked with sleep and cognitive difficulties. This result suggests that this specific dimension of CRF could interact with the other symptoms of the PNSC differently than the four other dimensions of CRF. As mental fatigue refers to cognitive difficulties, it is logical that it is particularly linked with the cognitive symptoms investigated in this study. The link we found between mental fatigue and sleep difficulties is in line with a recent study showing that sleep complaints (e.g., poor sleep quality, shorter time asleep, more wakes after sleep onset, lower sleep efficiency) were higher in patients with objective cancer‐related cognitive impairment [49]. Finally, cognitive difficulties, and thus mental fatigue, are known to be directly linked to other factors than comorbid symptoms. For examples, the cancer itself can impair cognitive function through increased systemic inflammation, as well as the oncological treatment received (e.g., chemotherapy, radiation therapy, hormonal therapy, immunotherapy), which can severely impact cognitive function at short and long terms through various mechanisms [50, 51]. The link between sleep difficulties and cognitive difficulties also seems to be particularly high in patients receiving hormonal treatment [49, 52]. In light of these studies, it is thus understandable that mental fatigue was not particularly associated with the core symptom of the network (i.e., depression) in our study. Our results also highlighted a difference in the centrality of each dimension of fatigue in their respective networks: mental and physical fatigues have a higher strength than the other dimensions. This suggests that these two dimensions are more directly connected to the other symptoms of their networks than the other CRF dimensions (i.e., general fatigue, lack of activities, and lack of motivation). Finally, our results highlighted a “Domino Effect” between different symptoms. This suggests that variations in depression scores (i.e., the core symptom in all networks) could lead to variations in the fatigue dimensions (except mental fatigue), as well as in all the other symptoms of the networks, directly and indirectly. This emphasizes the strong connections between symptoms from the PNSC. In this regard, some authors explained that the relationship between depression and fatigue does not seem to be bidirectional, as is generally believed [53]. They point out that one of the few studies that have investigated this relationship bidirectionally in oncology [54] showed that CRF predicted depressive mood better than depressive mood predicted CRF. This underlines the importance of investigating these symptoms and their interactions in oncology, particularly with a view to proposing intervention programs to improve the quality of life of patients with cancer.

### 3.1 Limitations

This study has a few limitations. First, it is a secondary analysis based on data from two distinct projects [33, 34]. These studies were not originally designed to assess symptom clusters through network analyses. This could have led to some biases in the selection of our participants (e.g., participant willing to participate in a mind–body intervention; meeting inclusion criteria that are particularly relevant in the context of interventional studies), limiting the generalization of our results. Second, the sample of this study consisted exclusively of females (breast cancer survivors), further limiting the generalizability of these results. In addition, the study faced a small sample size. Although network analyses are relatively recent and lack a consensus on the calculation of the required sample size, most of them included at least 250 participants [12, 27, 30]. However, two major studies in the field included 190 [11] and 172 [28] participants respectively, supporting the rationale for our analyses. Third, the covariates (e.g., age, time since diagnosis, possible comorbid conditions) were not controlled in the present analyses. Another limitation relies on the retrospective nature of the study and on the network analysis itself, which do not allow to infer causality relationships between different symptoms. Finally, the use of the HADS could represent a limitation and a potential bias in our study. While this scale is widely used and validated in various patient populations, including cancer patients, one of the main criticisms of the HADS is that some of its depression items may also measure aspects of adjustment rather than specifically capturing clinical depression [55, 56]. For example, items like “I feel as if I am slowed down,” “I still enjoy the things I used to enjoy,” and “I can laugh and see the funny side of things” may reflect a patient's response to the challenges of coping with cancer, rather than solely measuring the presence of clinical depression. A meta‐analysis suggests that the HADS is not recommended as a case‐finding instrument for identifying depression, anxiety, or distress in cancer settings, but is rather a screening tool allowing to detect depressive or anxious presymptomatology, or more generally adjustment difficulties [57]. Thus, the use of the HADS may have influenced our results. Questionnaires specific to depression (e.g., the Beck Depression Inventory [58]) and to anxiety (e.g., the State–Trait Anxiety Inventory [59]) could be used in future studies on the PNSC.

### 3.2 Implications for Futures Research

Depression and CRF are well established as strong predictors of breast cancer patients' quality of life [60, 61]. Understanding symptom clusters holds promise, as it enables proactive planning for comprehensive symptom management. Therefore, it is crucial to propose intervention programs aimed at reducing CRF that not only target this symptom, but also address its underlying causes and close associations with other symptoms within the cluster, such as depression. Network analyses offer a valuable approach to identify these interconnected processes since symptoms cannot be fully understood without considering their dependencies on other symptoms. This approach allows us to align with the clinical reality of the patients and develop appropriate management strategies. Future research should extend these findings to other cancer diagnoses to demonstrate the need for personalized intervention programs tailored to meet specific patient needs. Additionally, exploring temporal network analyses appears equally relevant, as cancer is a chronic disease that undergoes numerous changes over time. Psychological processes may significantly differ at the beginning or end of treatment, or during recurrence. This suggests the need for interventions that are tailored and adapted to the unique situation and time of care for each patient.

## 4 Conclusion

In conclusion, depression emerges as the core symptom in all estimated networks and showed strong associations with every dimension of fatigue, except mental fatigue. Our results confirmed the links existing between the symptoms of the PNSC (i.e., CRF, pain, emotional distress, sleep, and cognitive difficulties). However, they also pointed out some specificities of this cluster according to the CRF dimension considered, especially mental fatigue. In this way, our results strongly support the multidimensional aspect of CRF. Therefore, when constructing intervention programs to reduce patients' CRF, it is crucial not only to target CRF itself, but also to address the whole cluster of closely related symptoms. By doing so, we can optimize the effectiveness of such interventions and comprehensively improve the well‐being of patients.

## Author Contributions

**Louise Baussard:** conceptualization (lead), methodology (supporting), project administration (supporting), supervision (lead), writing – original draft (lead), writing – review and editing (lead). **Marie Ernst:** conceptualization (supporting), data curation (lead), formal analysis (lead), methodology (lead), software (lead), supervision (lead), writing – original draft (supporting), writing – review and editing (lead). **Anh Diep:** conceptualization (supporting), data curation (supporting), formal analysis (supporting), methodology (lead), software (supporting), writing – original draft (supporting), writing – review and editing (lead). **Guy Jerusalem:** conceptualization (supporting), project administration (supporting), supervision (supporting), writing – review and editing (equal). **Audrey Vanhaudenhuyse:** conceptualization (supporting), funding acquisition (lead), supervision (supporting), writing – review and editing (equal). **Nolwenn Marie:** investigation (lead), writing – review and editing (equal). **Isabelle Bragard:** conceptualization (supporting), funding acquisition (equal), writing – review and editing (equal). **Marie‐Elisabeth Faymonville:** conceptualization (supporting), writing – review and editing (supporting). **Olivia Gosseries:** conceptualization (supporting), funding acquisition (lead), supervision (supporting), writing – review and editing (equal). **Charlotte Grégoire:** conceptualization (lead), data curation (lead), funding acquisition (lead), investigation (lead), methodology (equal), project administration (lead), supervision (lead), writing – original draft (lead), writing – review and editing (lead).

## Ethics Statement

All procedures performed in this study were in accordance with the ethical standards of the institutional and national research committee and with the 1964 Helsinki Declaration and its later amendments or comparable ethical standards. The two studies involved in this article were approved by the Institutional Ethics Board “Hospital‐Faculty Ethics Committee of Liège” (Nos. B707201630321 and B7072020000081), with each participant providing written consent.

## Consent

The authors have nothing to report.

## Conflicts of Interest

The authors declare no conflicts of interest.

## Supporting information


Data S1.

## Data Availability

The full protocol and dataset of this study are available upon reasonable request. Please contact the corresponding author (ch.gregoire@uliege.be).
