# Supplementary material for: Network Analyses Applied to the Dimensions of Cancer‐Related Fatigue in Women With Breast Cancer
Source: Cancer Med. 2024 Oct 10;13(19):e70268. doi: 10.1002/cam4.70268 (PMC11465027; doi:10.1002/cam4.70268)

**Supplementary material**

**Table 1.** Associations (weights) between the different variables considered in the five sparse networks.

|  | **Sparse network - Weights** | Pain | HADS Anxiety | HADS Depression | MFI Fatigue dimension (i.e., the one considered in the network) | ISI Insomnia | FACT-Cog PCI | FACT-Cog OTH | FACT-Cog PCA | C FACT-Cog QQL | CERQ Adaptive Regulation | CERQ Non-Adaptive Regulation | MAC Positive adjustment |
| --- | --- | --- | --- | --- | --- | --- | --- | --- | --- | --- | --- | --- | --- |
| **General Fatigue network** | **HADS Anxiety** | 0 |  |  |  |  |  |  |  |  |  |  |  |
|  | **HADS Depression** | 0.025 | 0.226 |  |  |  |  |  |  |  |  |  |  |
|  | **MFI General Fatigue** | 0.076 | 0 | 0.159 |  |  |  |  |  |  |  |  |  |
|  | **ISI Insomnia** | 0 | 0.136 | 0 | 0.085 |  |  |  |  |  |  |  |  |
|  | **FACT-Cog PCI** | 0 | -0.059 | 0 | 0 | 0 |  |  |  |  |  |  |  |
|  | **FACT-Cog OTH** | 0 | -0.050 | -0.082 | 0 | 0 | 0.244 |  |  |  |  |  |  |
|  | **FACT-Cog PCA** | 0 | -0.005 | -0.152 | -0.046 | 0 | 0.497 | 0.088 |  |  |  |  |  |
|  | **FACT-Cog QQL** | -0.019 | 0 | -0.111 | -0.118 | -0.160 | 0.336 | 0 | 0.053 |  |  |  |  |
|  | **CERQ Adaptive Regulation** | 0 | -0.014 | -0.114 | 0 | 0 | 0 | 0 | 0 | 0 |  |  |  |
|  | **CERQ Non-Adaptive Regulation** | 0.076 | 0.120 | 0 | 0 | 0.068 | -0.007 | -0.004 | 0 | -0.023 | 0 |  |  |
|  | **MAC Positive adjustment** | 0 | -0.014 | -0.106 | -0.027 | 0 | 0 | 0 | 0.081 | 0 | 0.361 | 0 |  |
|  | **MAC Negative Adjustment** | 0 | 0.235 | 0.260 | 0 | 0.056 | -0.017 | -0.007 | 0 | 0 | -0.079 | 0.256 | 0 |
| **Physical Fatigue network** | **HADS Anxiety** | 0 |  |  |  |  |  |  |  |  |  |  |  |
|  | **HADS Depression** | 0.011 | 0.229 |  |  |  |  |  |  |  |  |  |  |
|  | **MFI Physical Fatigue** | 0.154 | 0 | 0.204 |  |  |  |  |  |  |  |  |  |
|  | **ISI Insomnia** | 0.020 | 0.151 | 0 | 0.032 |  |  |  |  |  |  |  |  |
|  | **FACT-Cog PCI** | 0 | -0.055 | 0 | 0 | 0 |  |  |  |  |  |  |  |
|  | **FACT-Cog OTH** | -0.017 | -0.055 | -0.078 | 0 | 0 | 0.257 |  |  |  |  |  |  |
|  | **FACT-Cog PCA** | 0 | 0 | -0.147 | -0.043 | 0 | 0.535 | 0.081 |  |  |  |  |  |
|  | **FACT-Cog QQL** | -0.018 | 0 | -0.110 | -0.138 | -0.185 | 0.355 | 0 | 0.037 |  |  |  |  |
|  | **CERQ Adaptive Regulation** | 0 | -0.025 | -0.129 | 0 | 0 | 0 | 0.016 | 0 | -0.051 |  |  |  |
|  | **CERQ Non-Adaptive Regulation** | 0.098 | 0.132 | 0 | 0 | 0.082 | -0.014 | -0.017 | 0 | -0.022 | 0.032 |  |  |
|  | **MAC Positive adjustment** | 0 | -0.030 | -0.115 | 0 | 0 | 0 | 0 | 0.099 | 0 | 0.397 | 0.044 |  |
|  | **MAC Negative Adjustment** | 0 | 0.237 | 0.243 | 0.094 | 0.057 | -0.004 | -0.006 | 0 | 0 | -0.105 | 0.281 | 0 |
| **Mental Fatigue network** | **HADS Anxiety** | 0 |  |  |  |  |  |  |  |  |  |  |  |
|  | **HADS Depression** | 0.052 | 0.235 |  |  |  |  |  |  |  |  |  |  |
|  | **MFI Mental Fatigue** | 0 | 0.008 | 0 |  |  |  |  |  |  |  |  |  |
|  | **ISI Insomnia** | 0.028 | 0.145 | 0 | 0.115 |  |  |  |  |  |  |  |  |
|  | **FACT-Cog PCI** | 0 | -0.049 | 0 | -0.108 | 0 |  |  |  |  |  |  |  |
|  | **FACT-Cog OTH** | -0.021 | -0.054 | -0.083 | 0 | 0 | 0.259 |  |  |  |  |  |  |
|  | **FACT-Cog PCA** | 0 | 0 | -0.142 | -0.300 | 0 | 0.470 | 0.074 |  |  |  |  |  |
|  | **FACT-Cog QQL** | -0.043 | 0 | -0.144 | -0.205 | -0.141 | 0.314 | 0 | 0 |  |  |  |  |
|  | **CERQ Adaptive Regulation** | 0 | -0.027 | -0.135 | 0 | 0 | 0 | 0.020 | 0 | -0.060 |  |  |  |
|  | **CERQ Non-Adaptive Regulation** | 0.106 | 0.135 | 0 | 0 | 0.082 | -0.011 | -0.018 | 0 | -0.025 | 0.039 |  |  |
|  | **MAC Positive adjustment** | 0 | -0.031 | -0.119 | 0 | 0 | 0 | 0 | 0.096 | 0 | 0.400 | 0.051 |  |
|  | **MAC Negative Adjustment** | 0 | 0.238 | 0.269 | 0.041 | 0.053 | 0 | -0.002 | 0 | 0 | -0.109 | 0.287 | 0 |
| **Lack of Motivation network** | **HADS Anxiety** | 0 |  |  |  |  |  |  |  |  |  |  |  |
|  | **HADS Depression** | 0.032 | 0.220 |  |  |  |  |  |  |  |  |  |  |
|  | **MFI Lack of Motivation** | 0 | 0.015 | 0.227 |  |  |  |  |  |  |  |  |  |
|  | **ISI Insomnia** | 0 | 0.137 | 0 | 0 |  |  |  |  |  |  |  |  |
|  | **FACT-Cog PCI** | 0 | -0.057 | 0 | -0.030 | 0 |  |  |  |  |  |  |  |
|  | **FACT-Cog OTH** | 0 | -0.048 | -0.079 | 0 | 0 | 0.240 |  |  |  |  |  |  |
|  | **FACT-Cog PCA** | 0 | -0.008 | -0.155 | 0 | 0 | 0.485 | 0.090 |  |  |  |  |  |
|  | **FACT-Cog QQL** | -0.023 | 0 | -0.129 | 0 | -0.171 | 0.331 | 0 | 0.065 |  |  |  |  |
|  | **CERQ Adaptive Regulation** | 0 | -0.014 | -0.112 | 0 | 0 | 0 | 0 | 0 | 0 |  |  |  |
|  | **CERQ Non-Adaptive Regulation** | 0.066 | 0.118 | 0 | 0 | 0.064 | -0.007 | 0 | 0 | -0.021 | 0 |  |  |
|  | **MAC Positive adjustment** | 0 | -0.007 | -0.080 | -0.106 | 0 | 0 | 0 | 0.076 | 0 | 0.347 | 0 |  |
|  | **MAC Negative Adjustment** | 0 | 0.227 | 0.223 | 0.124 | 0.058 | -0.013 | -0.006 | 0 | 0 | -0.070 | 0.248 | 0 |
| **Lack pf Activity network** | **HADS Anxiety** | 0 |  |  |  |  |  |  |  |  |  |  |  |
|  | **HADS Depression** | 0.044 | 0.231 |  |  |  |  |  |  |  |  |  |  |
|  | **MFI Lack of Activity** | 0 | 0 | 0.135 |  |  |  |  |  |  |  |  |  |
|  | **ISI Insomnia** | 0.014 | 0.146 | 0 | 0 |  |  |  |  |  |  |  |  |
|  | **FACT-Cog PCI** | 0 | -0.058 | 0 | 0 | 0 |  |  |  |  |  |  |  |
|  | **FACT-Cog OTH** | -0.007 | -0.052 | -0.081 | -0.015 | 0 | 0.248 |  |  |  |  |  |  |
|  | **FACT-Cog PCA** | 0 | -0.002 | -0.153 | -0.059 | 0 | 0.510 | 0.085 |  |  |  |  |  |
|  | **FACT-Cog QQL** | -0.035 | 0 | -0.102 | -0.188 | -0.179 | 0.338 | 0 | 0.041 |  |  |  |  |
|  | **CERQ Adaptive Regulation** | 0 | -0.016 | -0.117 | 0 | 0 | 0 | 0 | 0 | 0 |  |  |  |
|  | **CERQ Non-Adaptive Regulation** | 0.087 | 0.122 | 0 | 0 | 0.075 | -0.010 | -0.008 | 0 | -0.019 | 0 |  |  |
|  | **MAC Positive adjustment** | 0 | -0.017 | -0.113 | 0 | 0 | 0 | 0 | 0.087 | 0 | 0.374 | 0 |  |
|  | **MAC Negative Adjustment** | 0 | 0.236 | 0.255 | 0.054 | 0.061 | -0.010 | -0.007 | 0 | 0 | -0.082 | 0.262 | 0 |
| CERQ: Cognitive Emotion Regulation Questionnaire; HADS: Hospital Anxiety and Depression Scale; ISI: Insomnia Severity Index; MFI: Multidimensional Fatigue Inventoy; OTH: (comments from) OTHer (people about cognitive difficulties); PCA: Perceived Cognitive Abblities; PCI: Perceived Cognitive Impairment; QOL: (impact of cognitive difficulties on) Quality Of Life | | | | | | | | | | | | | |

**Table 2.** Description of centrality of sparse networks based on each dimension of CRF.

|  | Degree | | | | | Strength | | | | | Closeness (*1000) | | | | | Betweenness | | | | |
| --- | --- | --- | --- | --- | --- | --- | --- | --- | --- | --- | --- | --- | --- | --- | --- | --- | --- | --- | --- | --- |
|  | **General** | **Physical** | **Mental** | **Motivation** | **Activities** | **General** | **Physical** | **Mental** | **Motivation** | **Activities** | **General** | **Physical** | **Mental** | **Motivation** | **Activities** | **General** | **Physical** | **Mental** | **Motivation** | **Activities** |
| ***MFI Fatigue*** | *6* | *6* | *6* | *5* | *5* | *0.51* | *0.66* | *0.78* | *0.50* | *0.45* | *6.90* | *8.06* | *6.87* | *6.59* | *6.60* | *8* | *9* | *1* | *0* | *0* |
| Pain VAS | 4 | 6 | 5 | 3 | 5 | 0.20 | 0.32 | 0.25 | 0.12 | 0.19 | 3.75 | 5.41 | 3.96 | 2.96 | 3.49 | 0 | 0 | 0 | 0 | 0 |
| HADS Anxiety | 9 | 8 | 9 | **10** | 9 | 0.86 | 0.91 | 0.92 | 0.85 | 0.88 | 7.28 | 7.93 | 7.64 | 7.11 | 7.48 | 4 | 4 | 4 | 5 | 4 |
| HADS Depression | 9 | 9 | 8 | 9 | 9 | **1.24** | **1.27** | **1.18** | **1.26** | **1.23** | **9.26** | **10.3** | **9.57** | **8.80** | **9.22** | **31** | **26** | **25** | **36** | **28** |
| ISI Sleep | 5 | 6 | 6 | 4 | 5 | 0.51 | 0.53 | 0.56 | 0.43 | 0.47 | 6.11 | 6.82 | 6.52 | 5.66 | 6.44 | 0 | 1 | 0 | 0 | 1 |
| FACT-Cog PCI | 6 | 6 | 6 | 7 | 6 | **1.16** | **1.22** | **1.21** | **1.16** | **1.17** | 7.34 | 8.28 | 7.68 | 6.78 | 7.51 | 9 | 14 | 7 | 14 | 10 |
| FACT-Cog OTH | 6 | 8 | 8 | 5 | 8 | 0.47 | 0.53 | 0.53 | 0.46 | 0.50 | 5.59 | 6.11 | 5.95 | 5.17 | 5.65 | 0 | 0 | 0 | 0 | 0 |
| FACT-Cog PCA | 7 | 6 | 5 | 6 | 7 | 0.92 | 0.94 | 1.08 | 0.88 | 0.94 | 7.52 | 8.44 | 8.39 | 7.29 | 7.86 | 8 | 13 | 18 | 17 | 10 |
| FACT-Cog QOL | 7 | 8 | 7 | 6 | 7 | 0.82 | 0.92 | 0.93 | 0.74 | 0.90 | 7.22 | 8.12 | 8.13 | 6.75 | 7.01 | 7 | 9 | 2 | 3 | 7 |
| CERQ Adaptive | 4 | 7 | 7 | 4 | 4 | 0.57 | 0.76 | 0.79 | 0.54 | 0.59 | 5.31 | 6.33 | 6.19 | 5.19 | 5.43 | 0 | 2 | 3 | 6 | 0 |
| CERQ Non-Adaptive | 7 | 9 | 9 | 6 | 7 | 0.55 | 0.72 | 0.75 | 0.52 | 0.58 | 6.14 | 6.65 | 6.70 | 5.84 | 6.23 | 2 | 1 | 10 | 11 | 10 |
| MAC SPA | 5 | 5 | 5 | 5 | 4 | 0.59 | 0.68 | 0.70 | 0.62 | 0.59 | 5.41 | 6.32 | 6.13 | 4.96 | 5.64 | 2 | 3 | 4 | 1 | 3 |
| MAC SNA | 7 | 8 | 7 | 8 | 8 | 0.91 | 1.03 | 1.00 | 0.97 | 0.97 | 7.78 | 8.28 | 8.29 | 7.41 | 7.84 | 10 | 8 | 15 | 18 | 17 |
| CERQ: Cognitive Emotion Regulation Questionnaire; HADS: Hospital Anxiety and Depression Scale; ISI: Insomnia Severity Index; MFI: Multidimensional Fatigue Inventoy; OTH: (comments from) OTHer (people about cognitive difficulties); PCA: Perceived Cognitive Abblities; PCI: Perceived Cognitive Impairment; QOL: (impact of cognitive difficulties on) Quality Of Life; SNA: Summary Negative Adjustment ; SPA: Summary Positive Adjustment | | | | | | | | | | | | | | | | | | | | |

**Figure 1.** Results of parametric bootstraps: significant edges in the five networks.


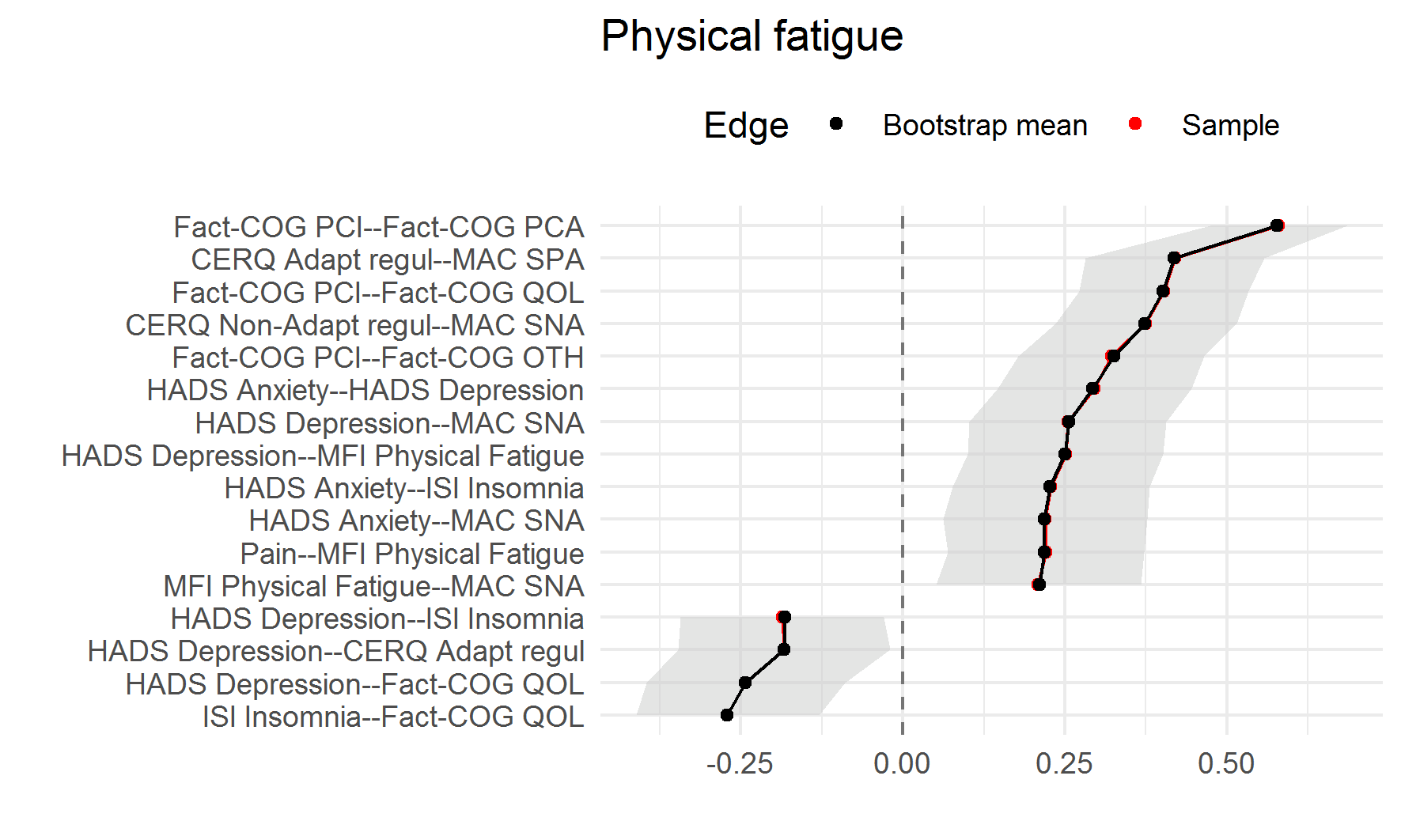

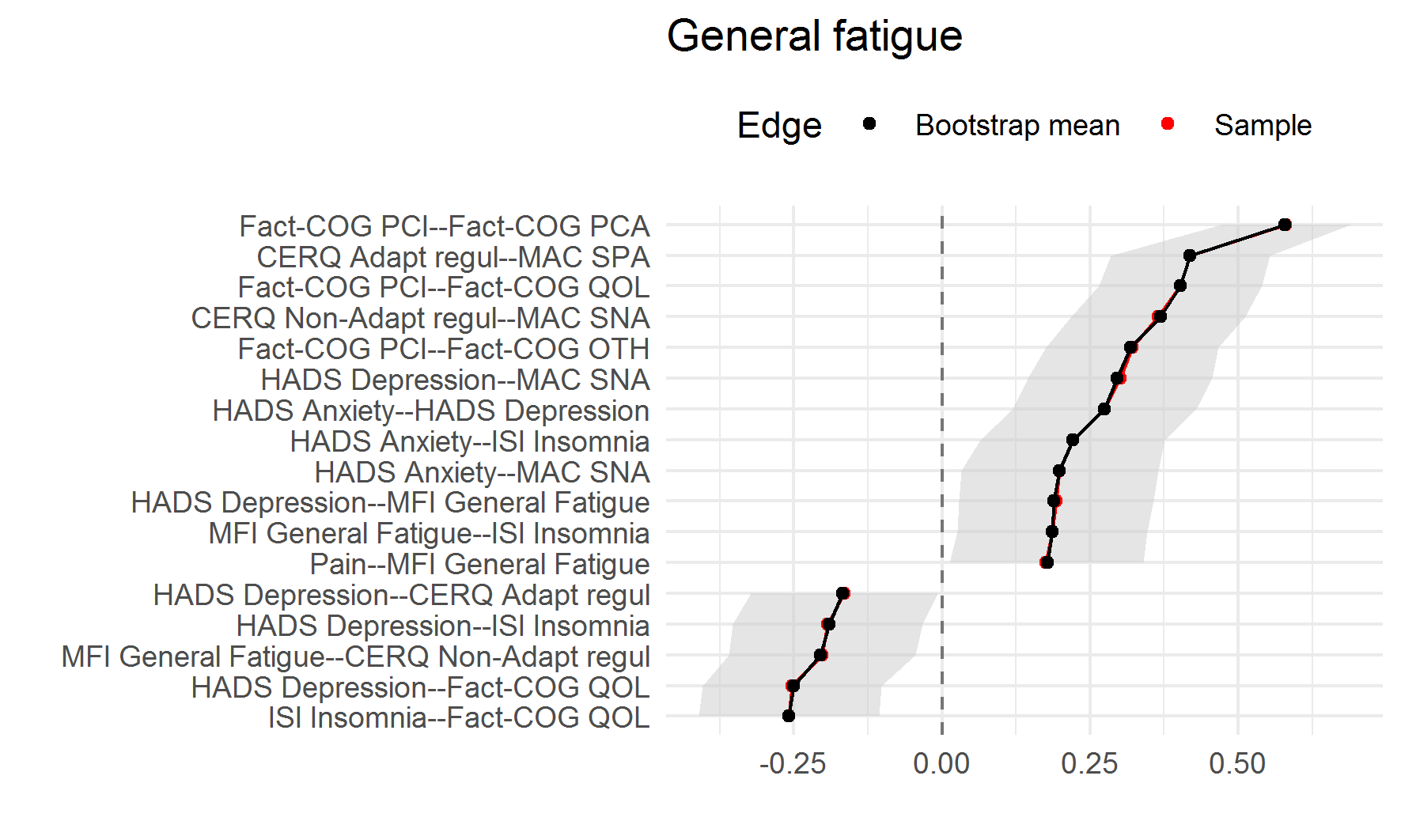

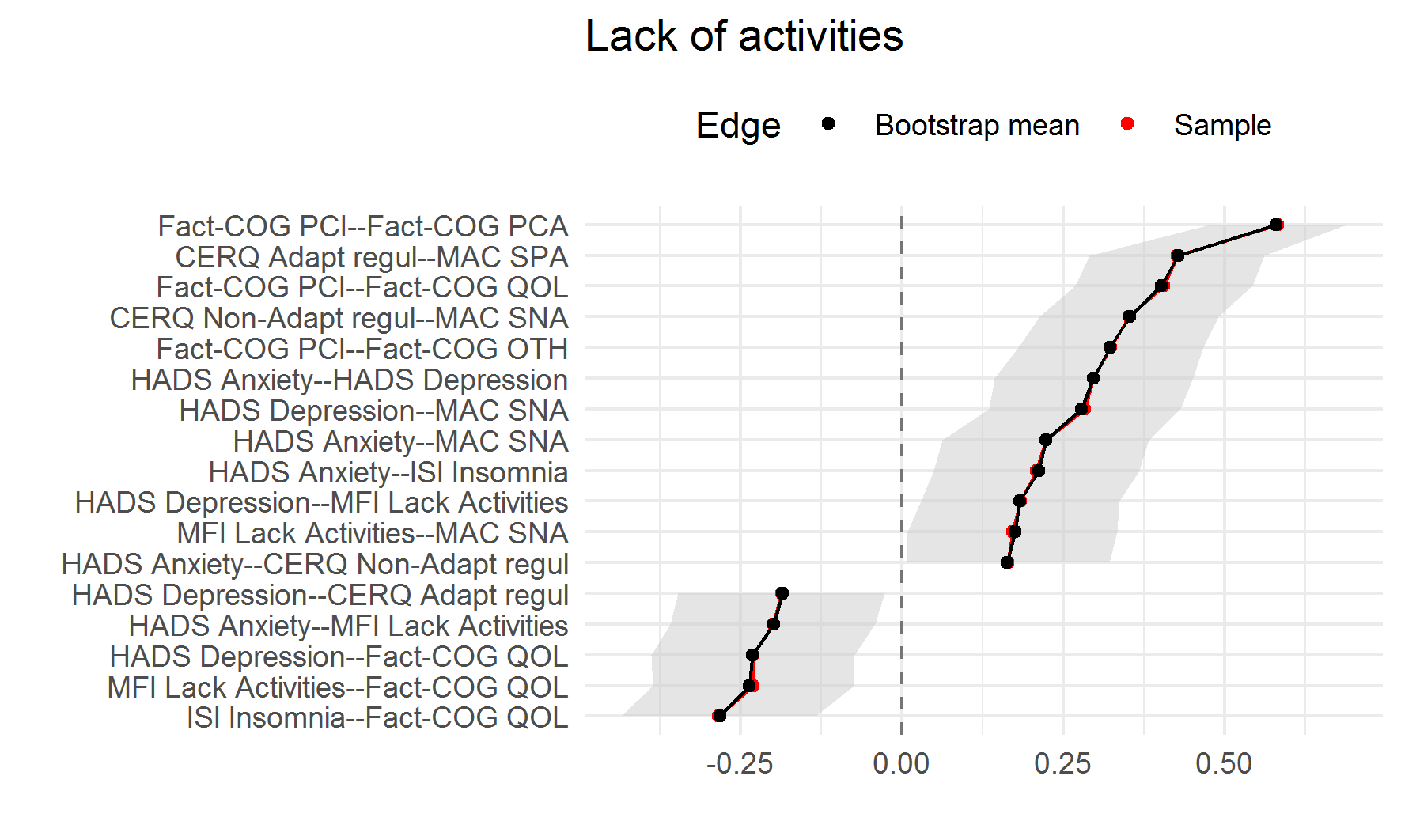

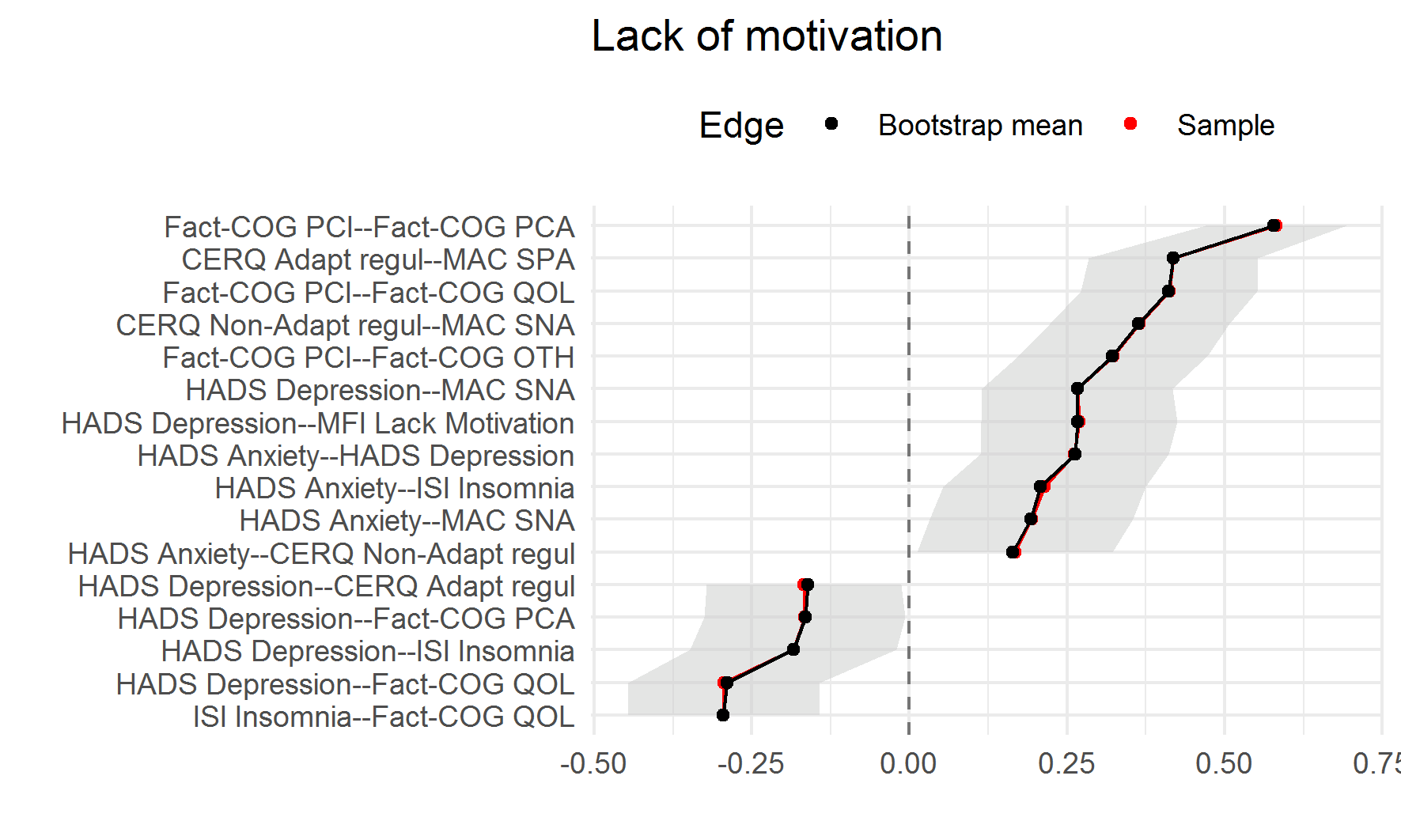

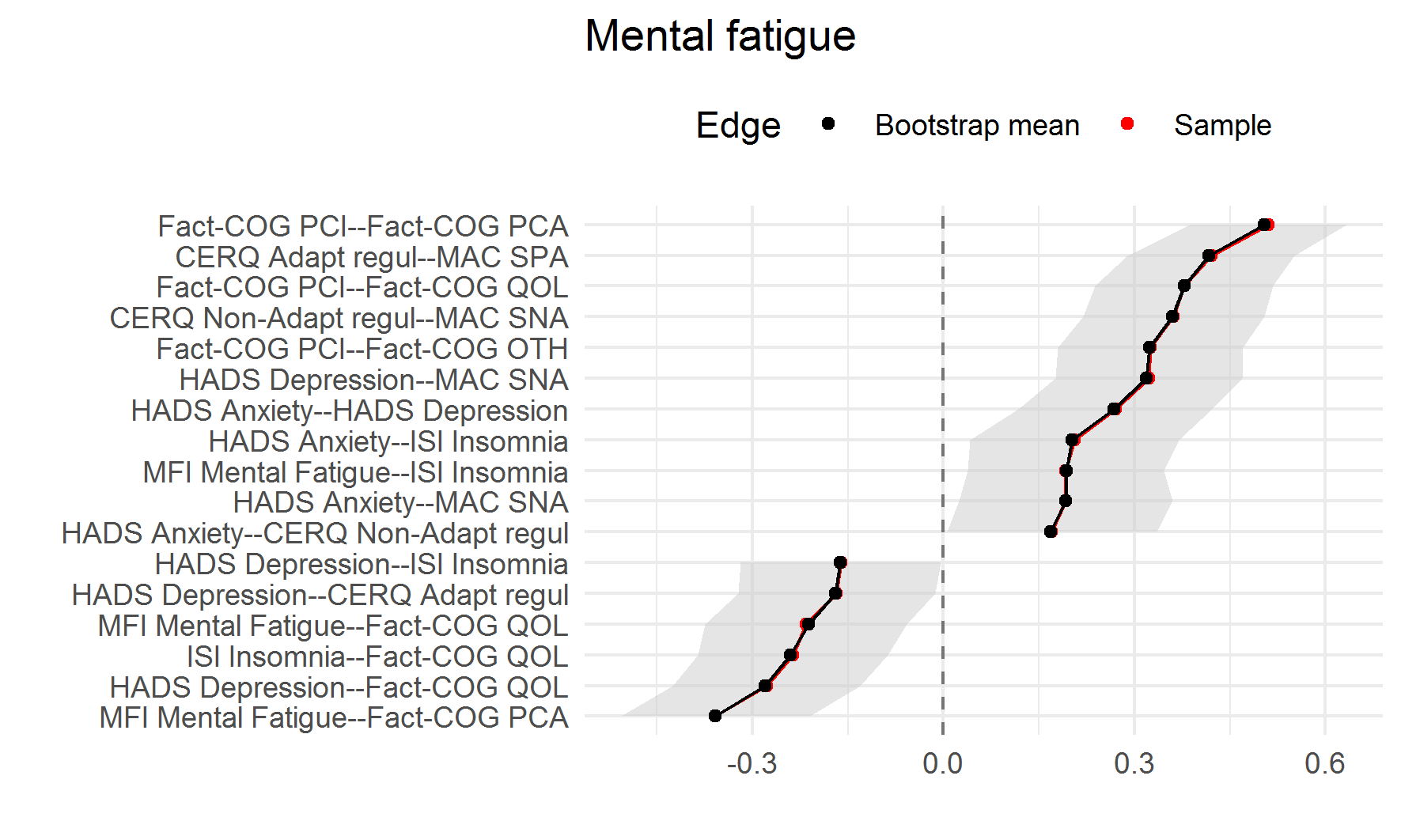


**Figure 2.** Results of parametric bootstraps: strength of each node in the five networks


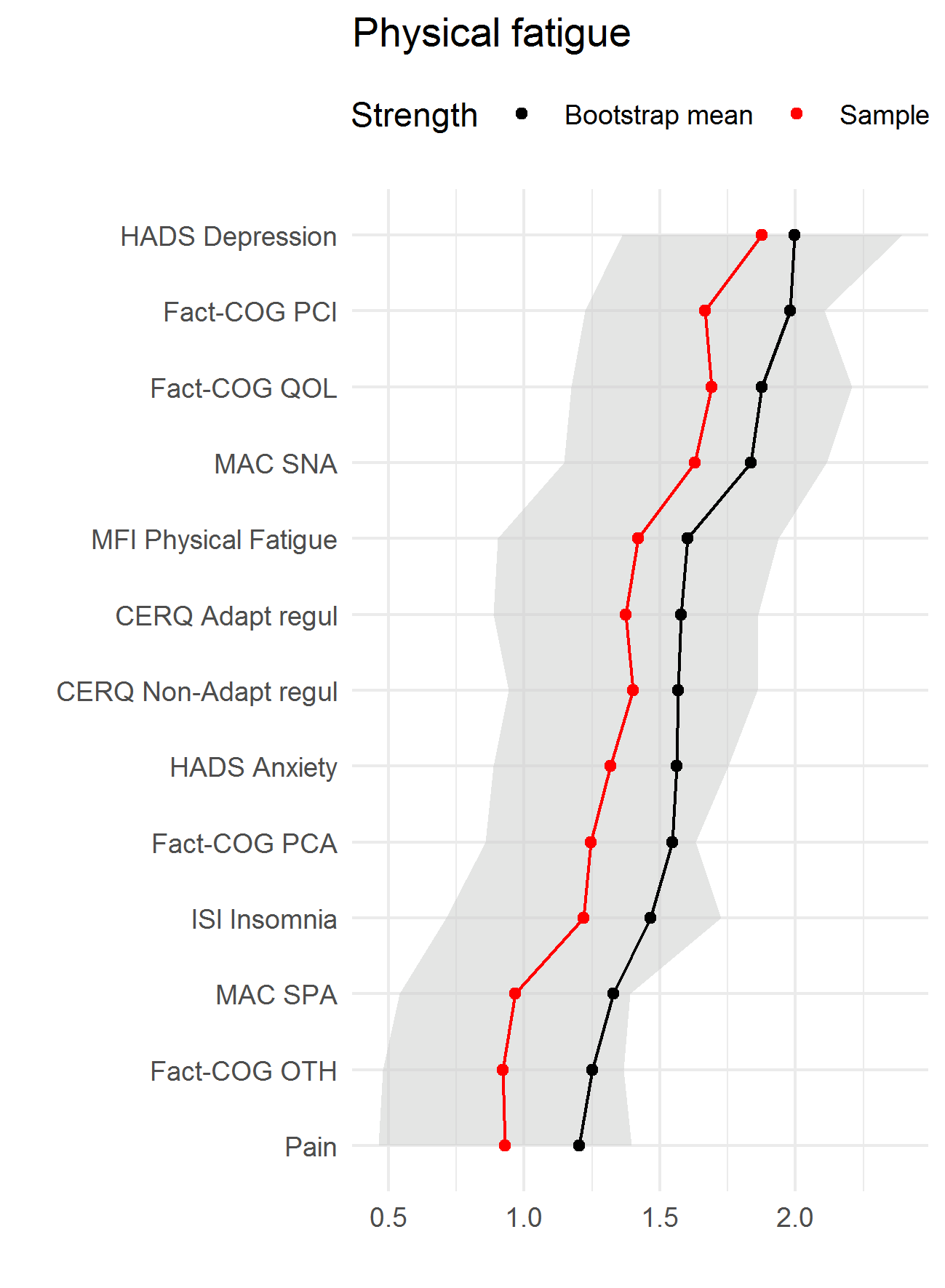

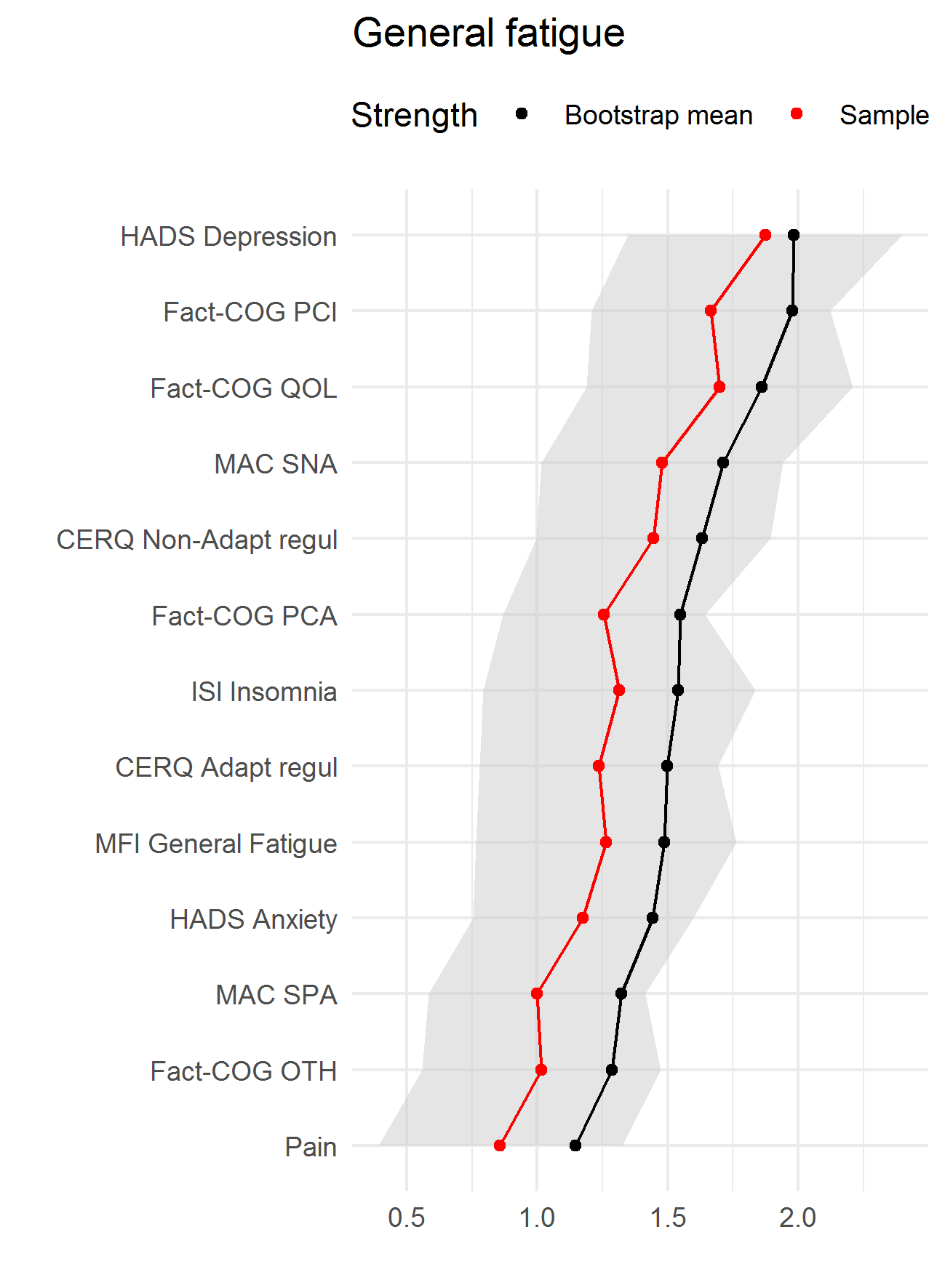

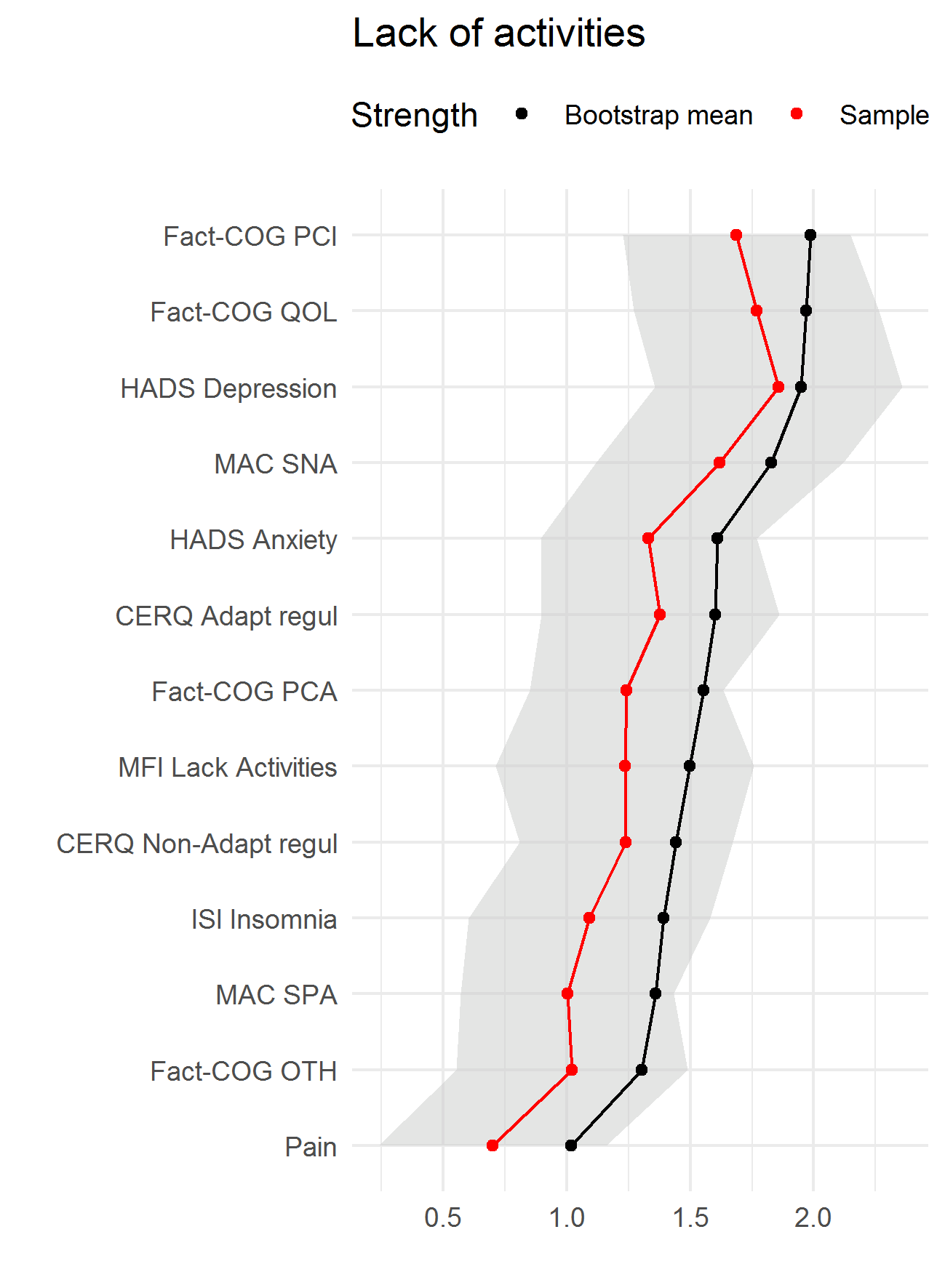

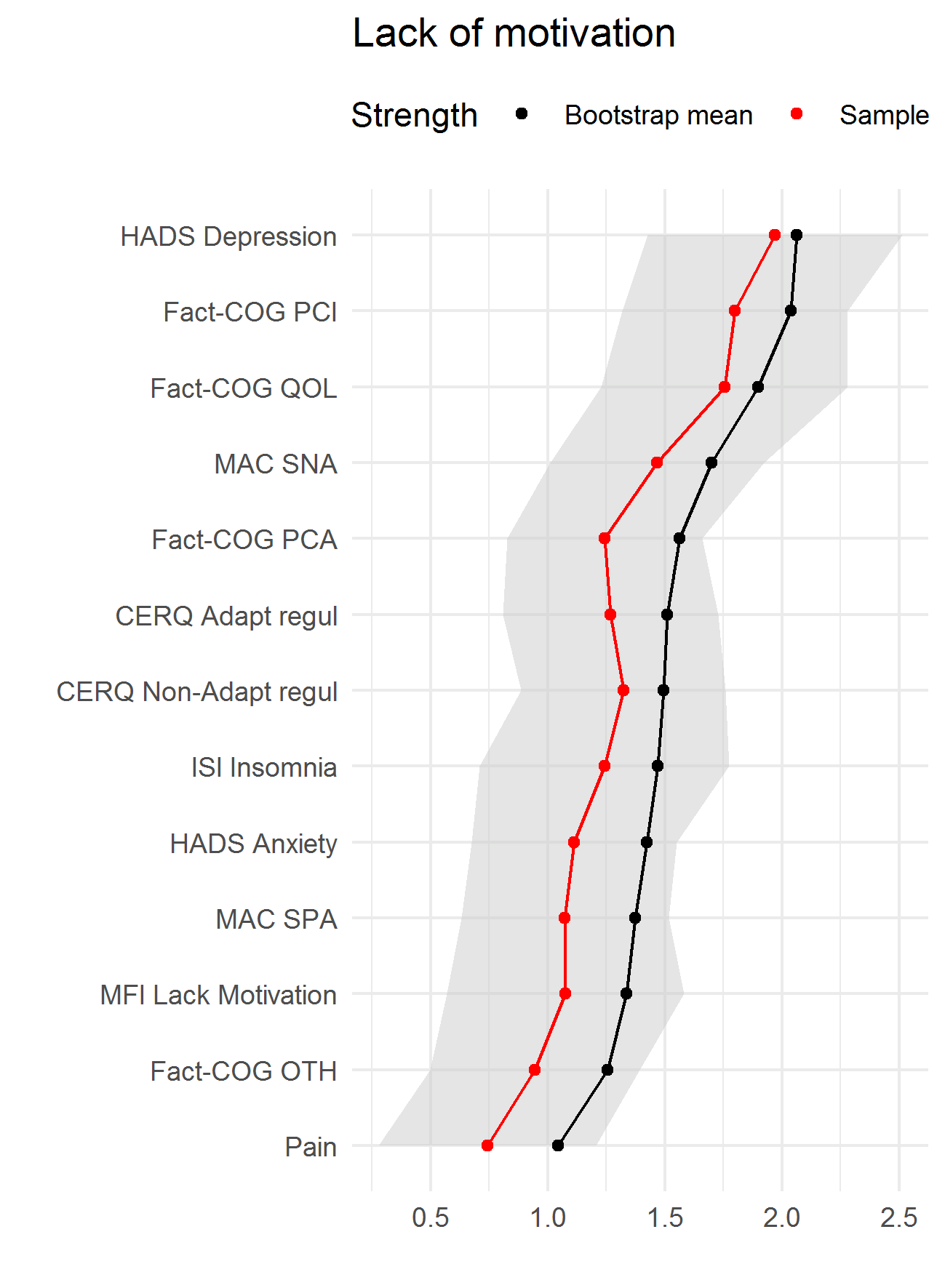

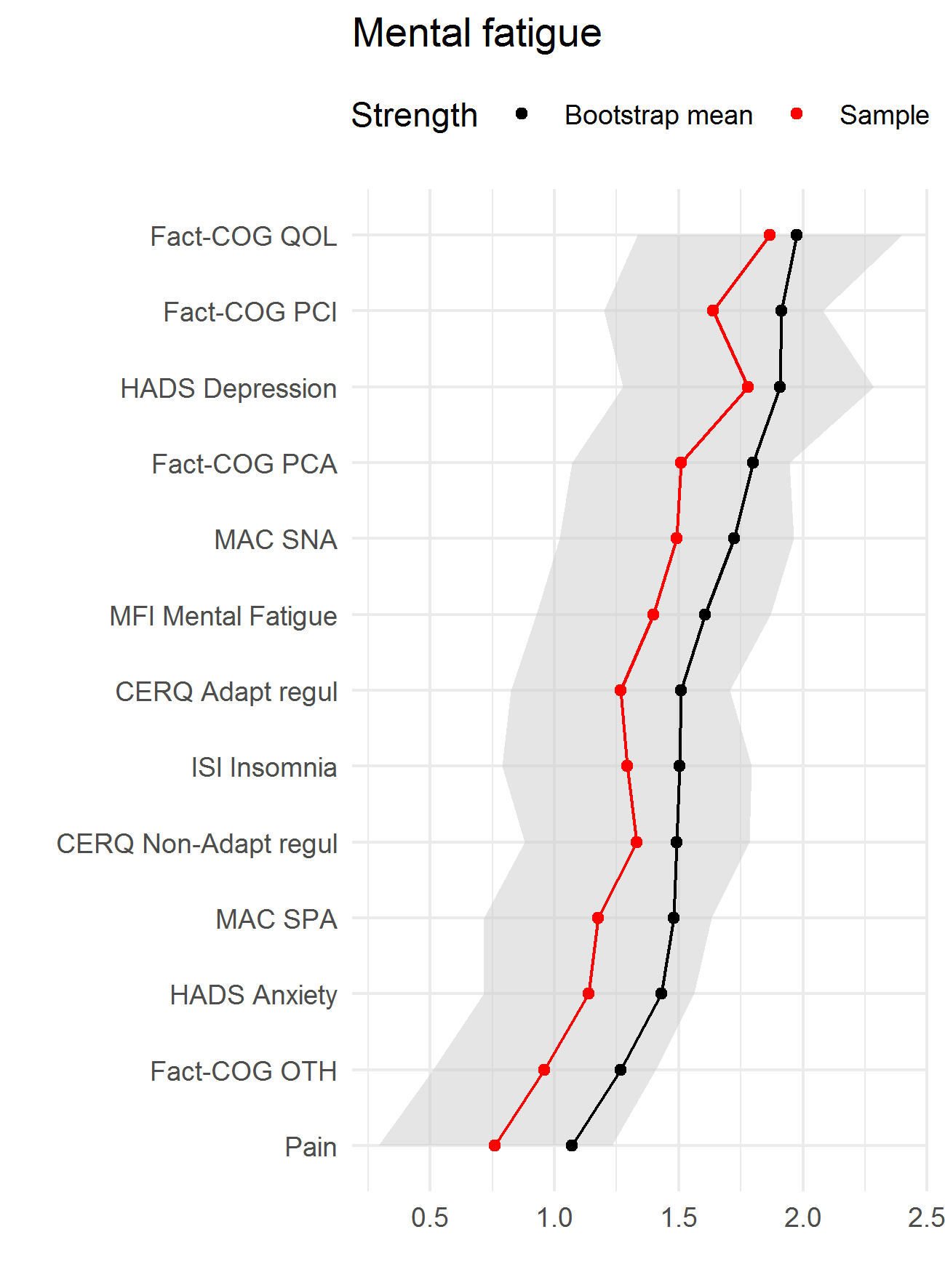

Supplement: Supplementary file 1 — Data S1. [file CAM4-13-e70268-s001.docx]
